# Self-efficacy in long-term prostate cancer survivors

**DOI:** 10.1016/j.ijchp.2025.100634

**Published:** 2025-10-15

**Authors:** Lilly J. Schmalbrock, Nils Kager, Florian Kirchhoff, Stefan Schiele, Andreas Dinkel, Jürgen E. Gschwend, Kathleen Herkommer

**Affiliations:** a Technical University of Munich, School of Medicine and Health, Department of Urology, TUM University Hospital, Germany; b Technical University of Munich, School of Medicine and Health, Department of Psychosomatic Medicine and Psychotherapy, TUM University Hospital, Germany

**Keywords:** Self-efficacy, Prostate cancer, Cancer survivor, Resilience

## Abstract

**Purpose:**

This study assessed self-efficacy (SE) among long-term prostate cancer survivors after radical prostatectomy and explored associated factors.

**Methods:**

As part of the nationwide project “Familial Prostate Cancer”, long-term prostate cancer survivors completed a follow-up survey, which included the validated General Self-Efficacy Short Scale (GSE-3). Sociodemographic, clinical, and psychosocial data were collected, including quality of life (QoL), symptoms of depression or anxiety, benefit finding, happiness, and perceived disease severity. Variables independently associated with SE were identified using a multiple linear regression analysis.

**Results:**

2534 prostate cancer survivors (mean age: 79.9 ± 6.4 years; mean follow-up: 18.3 ± 3.8 years post-RP) were included. The majority of men (97.0 %) were still undergoing regular follow-up. The mean SE score was 4.0 ± 0.7 (possible range 1–5). Lower SE was observed in men who were older, had lower educational level, poorer subjective economic status, another malignancy during lifetime, and were currently under treatment (all *p* < 0.05). Additionally, lower SE was associated with poorer QoL, reduced benefit finding, lower happiness, higher levels of depression and anxiety symptoms, and higher perceived disease severity (all *p* < 0.01). The regression model revealed independent associations between lower SE and older age, lower educational status, lower QoL, lower happiness and higher level of anxiety (all *p* < 0.05).

**Conclusions:**

General self-efficacy was rather high among long-term prostate cancer survivors. Sociodemographic and psychological variables, but no clinical parameters, were independently associated with SE.

**Implications for Cancer Survivors:**

Implementing interventions aimed at enhancing SE during follow-up care – particularly among older survivors, those with lower educational level, and symptoms of anxiety – could improve SE and thus positively affect QoL.

## Introduction

Prostate cancer (PCa) is the most prevalent cancer among men in 112 countries, representing 15 % of all cancer diagnoses. As the global population continues to age, the incidence of PCa is expected to rise substantially, with annual new cases projected to grow from 1,4 million in 2020 to 2,9 million by 2040. (James et al., 2024) Advances in early detection and treatment have led to a substantial improvement in clinical outcomes and survival rates, expanding the population of cancer survivors (Narayan et al., 2020; Williams et al., 2022). As survival rates continue to rise, the focus has shifted toward survivorship and the long-term challenges faced by men living with and beyond PCa (Harrington et al., 2010; Narayan et al., 2020; Skolarus et al., 2014).

Many PCa survivors experience persistent symptom burden that negatively affects their overall well-being, contributing to psychological distress, maladaptive thought patterns, and reduced coping abilities. A cancer diagnosis and its subsequent treatment can have profound psychosocial and physical consequences, with many men experiencing distress, anxiety, depression, fear of disease progression and a decline in quality of life (Bernat et al., 2016; Harrington et al., 2010; Meissner et al., 2021; Sanda et al., 2008; Skolarus et al., 2014). Long-term effects, including urinary incontinence, sexual dysfunction, fatigue, and cognitive impairments, further complicate survivorship (Bernat et al., 2016; Harrington et al., 2010; Klorek et al., 2025; Resnick et al., 2013; Sanda et al., 2008). Therefore, cancer is increasingly recognized as a chronic, long-term condition (Harrington et al., 2010; Narayan et al., 2020). Facing these challenges, the American Cancer Society underscores the importance of self-management strategies to mitigate symptoms and enhance quality of life (QoL) in PCa survivors (Skolarus et al., 2014).

Self-efficacy (SE), a concept introduced by Bandura in 1977, refers to an individual's confidence in their ability to perform behaviors that lead to desired outcomes (Bandura, 1978). In the context of chronic disease management, SE is a key mechanism for achieving effective self-management (Lorig & Holman, 2003; Marks et al., 2005; Peters et al., 2019). Consequently, its assessment has been recommended as a fundamental component of patient-centered care in chronic diseases (Peters et al., 2019). Regarding PCa and SE, studies have shown that higher SE is associated with improved physical function, reduced fatigue, and enhanced emotional and social well-being, particularly during the first year following treatment (Curtis et al., 2014; da Mata et al., 2015; Martín-Núñez et al., 2023, a, b; Mata et al., 2019). However, research on SE in long-term survivorship remains limited.

Given the growing population of PCa survivors, there is a critical need to further focus on this group and significantly enhance strategies to improve quality of life and long-term health outcomes. This study focuses on SE as a key component of this effort, aiming to assess the level of SE among long-term PCa survivors and to identify associated sociodemographic, clinical, and psychosocial factors.

## Method

### Study design and procedure

This analysis is part of the German nationwide research project “Familial Prostate Cancer”, which has prospectively recruited prostate cancer (PCa) patients since 1993. Detailed descriptions of the project are provided in previous publications (Dinkel et al., 2014; Paiss et al., 2003). In brief, participants complete annual questionnaires covering clinical, sociodemographic, and psychosocial information, with additional clinical data gathered from treating urologists. The present study analyzed cross-sectional data from the 2022 follow-up questionnaire. The project was approved by the ethics committee of the Technical University of Munich, and informed consent was obtained from all participants.

### Measures

#### Sociodemographic and clinical data

Sociodemographic parameters included partnership status, children, education level, and subjective economic status. Clinical parameters included age at survey, time since radical prostatectomy (RP), family history of PCa (yes/no), history of other malignancies, biochemical recurrence (PSA ≥ 0.2 ng/ml, categorized as before 2022, at the time of survey, or no), discontinuation of PSA follow-up, and ongoing PCa treatment at the time of the survey.

#### Self-efficacy

Self-efficacy was assessed using the German version of the General Self-Efficacy Short Scale (GSE-3, originally called ASKU in German), developed by Beierlein et al. (Beierlein et al., 2013). The GSE-3 is a unidimensional scale with three items: “I can rely on my own abilities in difficult situations”, “I am able to solve most problems on my own”, “I can usually solve even challenging and complex tasks well” [24]. Each item is rated on a five-point Likert scale from 1 ("does not apply at all") to 5 ("fully applies"). This standardized scale has been used in numerous studies (e.g. Pichler et al., 2022; Weidner et al., 2020) and has been adapted into other languages (Décieux et al., 2020; Pichler et al., 2022; Weidner et al., 2020). A mean scale score is computed, with higher scores indicating higher self-efficacy. Cronbach's alpha in the current sample was high (α = 0.89).

#### Quality of life (QoL, QL-2)

Quality of life was assessed using the German version of the items 29 and 30 from the EORTC QLQ-C30, focusing on global health and overall quality of life (QL-2) according to the EORTC QLQ-C30 Scoring Manual (Aaronson et al., 1993; Scott, 2008). Responses were rated on a 7-point Likert scale (1 = very poor, 7 = excellent) and transformed into a composite score (0–100). Higher scores reflect better QoL, with a score of ≥70 indicating good QoL and <70 indicating poor QoL (Aaronson et al., 1993; Scott, 2008).

#### Benefit finding

Benefit finding was assessed using one item (item 17) of the Benefit Finding Scale (BFS) adapted in German: “My prostate cancer has helped me become more focused on priorities, with a deeper sense of purpose in life” (Antoni et al., 2001; Mohamed NE, 2004). Responses were rated on a five-point scale (1 = not at all, 5 = extremely), categorized as 'low benefit finding' (responses 1–3) or high benefit finding' (responses 4–5) (Jahnen et al., 2023; Lassmann et al., 2021).

### Happiness

Happiness was assessed with the item: “How much of the time during the past month did you feel happy?” (Ryff & Keyes, 1995). Responses were given on a four-point scale (1 = none, 4 = all), categorized as 'low well-being' (responses 1–2) or 'high well-being' (responses 3–4).

#### Depressive and anxiety symptom

Symptoms of depression and anxiety were assessed using the German versions of the two-item Patient Health Questionnaire (PHQ-2) and the two-item Generalized Anxiety Disorder scale (GAD-2) (Kroenke et al., 2009). Each scale is rated on a 4-point Likert scale (0–3), with a sum score of ≥3 indicating clinical levels of depressive or anxious symptoms (Kroenke et al., 2009). Cronbach’s alpha values for the PHQ-2 and GAD-2 were 0.71 and 0.77, respectively, demonstrating satisfactory internal consistency in this sample.

#### Perceived severity of disease

Perceived severity of PCa was assessed with the item: “Having had prostate cancer is one of the worst things that happened to me in my life” (adapted from Vadaparampil et al. (2004)). Responses were given on a 4-point Likert scale from “strongly disagree” (1) to “strongly agree” (4). Scores were categorized as 'low perceived severity' (responses 1–2) or 'high perceived severity' (responses 3–4) (Vadaparampil et al., 2004).

### Statistical analysis

Descriptive statistics summarized the sociodemographic and clinical parameter of the cohort. Differences in SE across sociodemographic, clinical, and psychosocial parameters were assessed using Wilcoxon Two-Sample tests or Kruskal-Wallis tests. A multiple linear regression model was used, treating SE as criterion variable. To identify variables independently associated with SE, the following parameters were included in the linear regression analysis: age, education level, partnership status, subjective economic situation, time since RP, history of secondary cancer, biochemical recurrence (BCR), ongoing therapy, QoL, benefit finding, happiness, depression, anxiety, and perceived severity of the PCa condition. Men with missing data for any of the listed parameters were excluded from the linear regression analysis. Analyses were performed using SAS 9.4 (SAS Institute, Cary, NC, USA). A two-tailed p-value < 0.05 was considered statistically significant.

## Results

### Patient characteristics

**Fig. 1:**
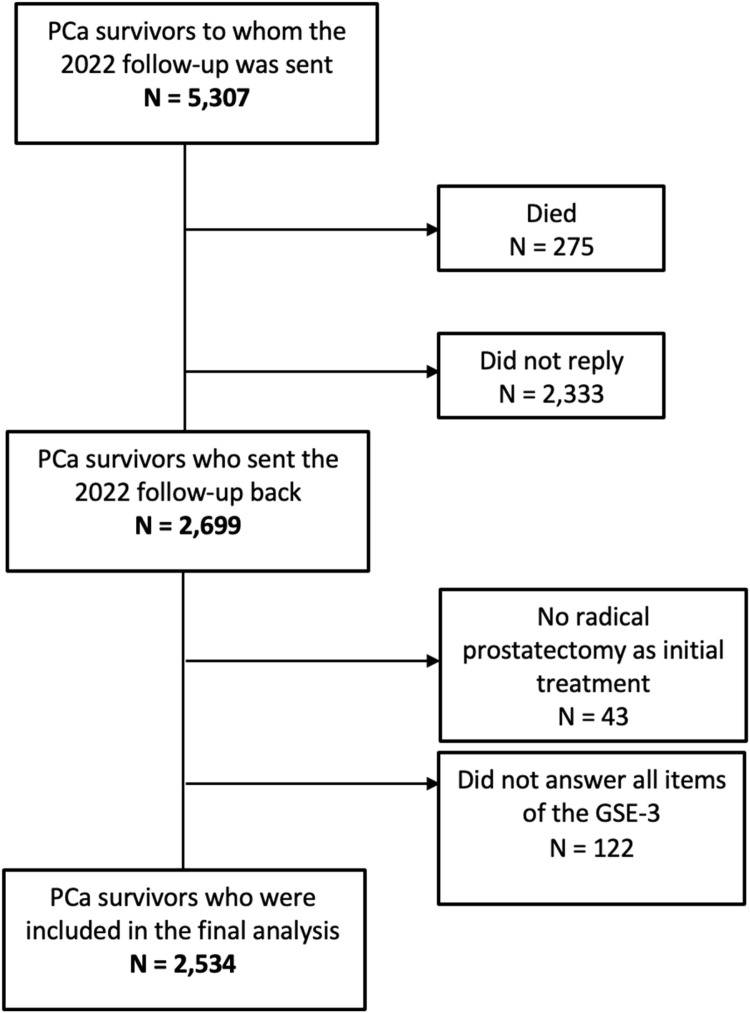
Flowchart detailing inclusion and exclusion criteria leading to the final sample of PCa survivors included in the analysis.

By January 2023, 2699 PCa survivors had returned the annually questionnaire 2022. Of these, only men who had undergone radical prostatectomy as primary treatment and who completed all three items assessing SE were included. (Fig. 1)

Thus, data from 2534 PCa survivors with a mean age of 79.9 ± 6,4 years and a mean follow-up-time of 18.3 ± 3.8 years since RP were analyzed. Most were in a partnership (87.9 %) and had children (88.7 %). 61.9 % reported intermediate or high educational level and 75.0 % perceived their economic status as good. 11.1 % of men were under therapy at the time of survey. 38.1 % had a current or past biochemical recurrence (BCR), and 13.1 % reported another malignancy during their lifetime. The mean QoL score was 68.2 ± 19.2. Clinical levels of anxiety or depression were present in 11.0 % and 13.8 % of participants, respectively. The majority of men (97.0 %) were still undergoing regular follow-up (Table 1).

**Table 1:** Baseline Characteristics of the study sample (*N* = 2534) and differences in self-efficacy using the General Self-Efficacy Short Scale in relation to sociodemographic, clinical and psychosocial parameters.

Parameter	**%**	**n**	***M* ± SD**	**self-efficacy *M* ± SD**	**p-value**
**Sociodemographic parameter**					
Age at survey (years)			79.9 ± 6.4		
Age groups					.**001**
≤ 75	21.3	541		4.08 ± 0.59	
> 75 - 85	58.4	1480		4.00 ± 0.69	
>85	20.2	513		3.87 ± 0.83	
Partnership					.364
Yes	87.9	2003		4.01 ± 0.67	
No	12.1	276		3.94 ± 0.79	
Children					.615
Yes	88.7	2227		3.99 ± 0.71	
No	11.3	285		4.02 ± 0.66	
Educational level					**<0.001**
Low	38.1	935		3.90 ± 0.76	
Intermediate	17.1	420		4.00 ± 0.63	
High	44.8	1102		4.06 ± 0.66	
Self-perceived economic status					**<0.001**
Good	75.0	1720		4.07 ± 0.66	
Poor	25.0	574		3.81 ± 0.70	
**Clinical parameter**					
Time since radical prostatectomy (years)			18.3 ± 3.8		**.005**
≤ 5	17.3	437		4.05 ± 0.64	
> 15 – 20	50.3	1275		4.01 ± 0.69	
> 20	32.4	822		3.92 ± 0.75	
Family history of PCa					.532
Yes	41.6	1054		4.00 ± 0.69	
No	58.4	1480		3.98 ± 0.71	
Other malignancy during lifetime					**.009**
Yes	13.1	331		3.90 ± 0.74	
No	86.9	2203		4.00 ± 0.70	
Biochemical recurrence (BCR)					.093
Yes (not in 2022)	20.1	510		3.97 ± 0.73	
Yes (in 2022)	18.0	455		3.92 ± 0.76	
No	61.9	1569		4.01 ± 0.68	
Discontinued PSA follow-up					.874
Yes	3.0	75		3.96 ± 0.79	
No	97.0	2459		3.99 ± 0.70	
Ongoing treatment at survey					**.046**
Yes	11.1	280		3.90 ± 0.81	
No	88.9	2254		4.00 ± 0.69	
**Psychosocial parameter**					
Quality of life (QL-2)			68.2 ± 19.2		**<0.001**
Low (< 70)	54.8	1373		3.81 ± 0.72	
High (≥ 70)	45.2	1133		4.22 ± 0.60	
Benefit finding			2.5 ± 1.1		**<0.001**
Low	78.3	1932		3.96 ± 0.71	
High	21.7	535		4.09 ± 0.63	
Happiness			1.7 ± 0.7		**<0.001**
Low	40.1	991		3.74 ± 0.73	
High	59.9	1480		4.16 ± 0.62	
Depression (PHQ-2)			1.2 ± 1.3		**<0.001**
None (< 3)	86.2	2123		4.05 ± 0.67	
Positive (≥ 3)	13.8	339		3.65 ± 0.78	
Anxiety (GAD-2)			1.1 ± 1.2		**<0.001**
None (< 3)	89.0	2169		4.06 ± 0.66	
Positive (≥ 3)	11.0	268		3.52 ± 0.81	
Perceived severity of disease			2.6 ± 0.9		**.031**
Low	47.6	1186		4.02 ± 0.70	
High	52.4	1303		3.96 ± 0.70	

Note: PCa = prostate cancer, PHQ-2 = Patient Health Questionnaire, GAD-2 = Generalized Anxiety Disorder scale.

The mean SE score was 4.00 ± 0.70. Lower SE was observed in men who were older, had lower educational level, poorer self-perceived economic status, had another malignancy during lifetime, and were currently under treatment (all *p* < 0.05). Additionally, lower SE was associated with poorer QoL, reduced benefit finding, lower happiness, clinical depression and anxiety, and higher perceived disease severity (all *p* < 0.01) (Table 1).

All psychosocial variables showed low intercorrelations, with the exception of depression and anxiety, which correlated moderately. Furthermore, all variables were significantly correlated with SE, with QoL showing the highest association (*r* = 0.40, *p* < 0.001) (Table 2).

**Table 2:** Intercorrelation matrix (Spearman correlations) of the psychosocial parameters included in this analysis.

		1	2	3	4	5	6	7
1	Quality of Life	–	−0.04	.55	−0.54	−0.47	−0.11	.40
2	Benefit Finding		–	.04	.12	.15	.21	.04
3	Happiness			–	−0.48	−0.41	−0.13	.34
4	Depression				–	.66	.17	−0.34
5	Anxiety					–	.20	−0.35
6	Perceived Severity						–	−0.05
7	Self-efficacy							–

The multiple linear regression analysis revealed that lower SE was independently associated with higher age, lower educational level, lower QoL, lower well-being and more symptoms of anxiety. The included clinical variables were not significantly associated with self-efficacy and did not meaningfully increase the explained variance (Table 3). Overall, the model explained 21.1 % of the variance in self-efficacy (adj. R²).

**Table 3:** Results of the multiple linear regression analysis, of variables associated with self-efficacy (*n* = 1997).

**Parameter**	**B**	**SE B**	**Beta**	**p-value**
**Age at survey (years)**	−0.006	.002	−0.058	**.007**
Partnership Yes (Ref: No)	−0.059	.042	−0.029	.154
**Educational level** (Ref: Low)				
Intermediate	.046	.040	.026	.246
**High**	.107	.030	.080	**<0.001**
Self-perceived economic status (Ref: Good)				
Poor	−0.064	.033	−0.041	.052
Time since radical prostatectomy (years)	−0.006	.004	−0.032	.129
Other malignancy during lifetime Yes (Ref: No)	−0.005	.040	−0.003	.896
BCR* (Ref: No)				
Yes (but not 2022)	.013	.036	.008	.716
Yes (in 2022)	−0.002	.039	−0.001	.955
Ongoing treatment at survey Yes (Ref: No)	.035	.048	.016	.469
**Quality of life (QL-2)**	.006	.000	.168	**<0.001**
Benefit Finding	.004	.013	.006	.768
**Happiness**	.141	.024	.145	**<0.001**
Depression (PHQ-2)	−0.027	.016	−0.050	.093
**Anxiety (GAD-2)**	−0.100	.015	−0.183	**<0.001**
Perceived severity of disease	.018	.015	.025	.235

Note: All variables were entered as continuous measures, except where indicated.

⁎ Biochemical recurrence.

## Discussion

Prostate cancer (PCa) survivors represent a growing patient population. However, long-term support strategies addressing their physical, functional, and psycho-oncological needs remain insufficient (Skolarus et al., 2014). In other chronic diseases, such as hypertension and type 2 diabetes, self-efficacy (SE) has been identified as a crucial factor in enhancing QoL, well-being, and adherence to treatment regimens (Chen et al., 2023; Peters et al., 2019). In the context of PCa, SE has been poorly studied, with existing research primarily focusing on the first year post-treatment (Curtis et al., 2014; da Mata et al., 2015; Martín-Núñez et al., 2023, a, b; Mata et al., 2019; Weber et al., 2007). Studies addressing SE as an essential component of self-management and psychosocial adaptation in long-term PCa survivors are lacking.

This study provides the first assessment of SE in a large sample of >2500 long-term PCa survivors, with a mean follow-up of 18 years. To the best of our knowledge, no previous study has investigated SE in this population over such an extended period. Additionally, this study is the first to examine factors associated with SE in long-term PCa survivors. Given the increasing number of men living many years post initial treatment of PCa, this group requires special attention within the healthcare system.

The mean SE level observed in this study was generally high, suggesting that long-term PCa survivors have a relatively strong sense of confidence in their ability to cope with challenging situations in general. Similar SE levels have been reported in other studies using the GSE-3 evaluating SE in men with advanced PCa (Bugaj et al., 2023; Pichler et al., 2022). In the univariable analysis of this study, several factors including higher age, poorer economic status, another malignancy during lifetime, being under treatment or higher perceived disease severity were associated with lower SE.

While the association of PCa-specific factors such as being under current treatment, or a perceived disease severity lost significance in the multivariable analysis, demographic factors including higher age and lower educational level remained to be significantly associated with lower SE. This finding may be explained by the fact that long-term survivors may have already adapted to the stressors associated with their disease. Additionally, as in the general aging population, the daily lives of PCa survivors are often influenced more by comorbidities than by PCa-specific parameters (Klorek et al., 2025). Nevertheless, long-term PCa survivors remain a distinct group of aging men with a high symptom burden across multiple domains, often because of initial or subsequent PCa treatment.

Moreover, in multivariable analysis, consistent with previous research (da Mata et al., 2015; Hou et al., 2024; Kawaguchi et al., 2020; Martín-Núñez et al., 2023, a; Weber et al., 2007), lower SE was linked to decreased QoL, happiness or well-being, and more symptoms of anxiety, while higher SE was associated with better psychological outcomes. Moreover, lower SE has been associated with increased anxiety, which negatively impacts mental health. Anxiety reduction is known to support more effective coping strategies, enabling patients to manage stressors and long-term postoperative challenges more successfully (da Mata et al., 2015).

Previous research has demonstrated that PCa survivors with lower income and lower education tend to report poorer QoL compared to those with higher socioeconomic status (Ramsey et al., 2007; Shi et al., 2011). SE may act as a mediating factor in this relationship, as it has been shown to mediate the effects of social support and autonomy in other contexts (Wang et al., 2022; Warner et al., 2011). Moreover, controlled support group interventions have been associated with significant improvements in SE, emotional well-being, and disease-specific knowledge. Notably, older men and those with lower education levels appear to benefit the most from such interventions (Helgeson et al., 2006; Lepore et al., 2003).

Most studies assessing SE have focused on the immediate postoperative period, typically within one year after RP or initial treatment (da Mata et al., 2015; Hou et al., 2024; Kawaguchi et al., 2020; Martín-Núñez et al., 2023, a; Weber et al., 2007). Intervention studies in this context have shown that men receiving SE-enhancing interventions, or those with inherently higher SE, experience better QoL (Kawaguchi et al., 2020; Weber et al., 2007), healthier behaviors (Baskin et al., 2016; Williams & Rhodes, 2016), and improved psychological and physiological health maintenance (da Mata et al., 2015; Hou et al., 2024; Kawaguchi et al., 2020). For example, a study by Weber et al. demonstrated that a dyadic peer support intervention, designed to enhance SE through vicarious experience — one of the four initially defined primary sources of SE (mastery and vicarious experience, verbal persuasion, arousal state) —effectively improved both SE and QoL in men up to eight weeks post-RP (Weber et al., 2007).

The relatively modest explained variance of 21.1 % suggests that additional relevant factors influencing self-efficacy were not captured in the current model. Previous research indicates that psychosocial and contextual resources—such as autonomy and social support, including both positive and negative aspects, as well as disease knowledge—play a significant role in shaping self-efficacy and may be reciprocally influenced by it (Warner et al., 2011; Wu et al., 2025). Future studies should therefore incorporate these factors and conduct expanded analyses to better elucidate their impact in prostate cancer survivors.

Despite increasing recognition of the long-term consequences of PCa, research on survivorship remains relatively limited. Many survivors experience persistent effects, including urinary incontinence, sexual dysfunction, bowel issues, and psychosocial challenges (Bernat et al., 2016; Darwish-Yassine et al., 2014; Resnick et al., 2013; Sanda et al., 2008; Skolarus et al., 2014). Findings from a previous follow-up analysis from the German research project “Familial Prostate Cancer” suggest that the three most frequently reported medical problems were hypertension, back pain, and osteoarthritis. In contrast, urological issues related to cancer therapy were rarely identified as a problem, although they were perceived as impairing daily life (Klorek et al., 2025). Another study, with an average follow-up of nine years post-treatment, reported a high symptom burden across multiple domains (e.g., sexual dysfunction: 44.4 %, urinary issues: 14.4 %, reduced vitality: 12.7 %, bowel issues: 8.4 %, emotional distress: 7.6 %), with more than half of respondents (56 %) expressing a need for additional information (Bernat et al., 2016). These findings underscore the importance of assessing symptom burden, supporting self-management, and fostering SE beyond the first-year post-treatment. Unfortunately, data on functional status and comorbidities were not available for the present analysis, which must be mentioned as a limitation of this analysis. Moreover, in this study, general SE was assessed. Future studies may also include cancer-related SE (Huang et al., 2018; Manne et al., 2006). Including both general and cancer-related SE may provide more detailed insights into survivors’ coping abilities, facilitate the exploration of potential differences, and may contribute to the development of tailored intervention strategies.

From a clinical perspective, these findings underscore the need for structured and targeted survivorship care that extends beyond the acute treatment phase. Self-efficacy, a key psychological resource, can be strengthened through mastery experiences (e.g., achievable behavioural tasks), vicarious experiences or social modeling (e.g., peer or mentor interaction), verbal persuasion (e.g., positive reinforcement) (Bandura, 1978). These mechanisms can be addressed through established interventions, including cognitive-behavioural therapy, psychoeducational or peer-led support groups, and family-inclusive programs.

Despite a growing population of prostate cancer survivors, standardized follow-up programmes in general, but especially incorporating self-efficacy training remain rare. Addressing this gap is increasingly recognized as a clinical priority (Dunn et al., 2021; Harrington et al., 2010; Narayan et al., 2020). In particular, older prostate cancer survivors with lower self-efficacy may face increased psychological distress, reduced adherence to follow-up care, and poorer quality of life, potentially exacerbated by comorbidities, cognitive decline, social isolation, or limited familiarity with digital health resources. These factors may restrict their ability to effectively manage their health, underscoring the importance of tailored interventions for this group. One promising model is the *Prostate Cancer Patient Empowerment Program (PC-PEP)*, a structured 6-month intervention that has shown positive outcomes in a phase II randomized clinical trial. PC-PEP incorporates all three core self-efficacy mechanisms: it includes goal setting and progressive tasks (mastery experiences), weekly interaction with mentors and peers (social modeling), and daily motivational content (social persuasion). The program also leverages modern technologies, such as emails, SMS reminders, videos, and an app-connected biofeedback device, which help to reduce access barriers and sustain behavioral changes over time (Ilie et al., 2023; MacDonald et al., 2024). Such digital and multimodal interventions can be flexibly implemented in different care settings and tailored to individual patient needs. They also align with current recommendations to integrate psycho-oncological support into routine survivorship care (Dunn et al., 2021). In summary, our findings support the integration of self-efficacy–enhancing components into survivorship care for prostate cancer patients, particularly in long-term follow-up. Future efforts should focus on developing scalable, evidence-based programmes that can be tailored to the needs of diverse patient populations and embedded within routine clinical care.

## Conclusions

Self-efficacy levels were generally high among long-term prostate cancer survivors but lower in older individuals, those with lower education and symptoms of anxiety. Interventions to enhance SE could be particularly beneficial for these groups and should be integrated into outpatient follow-up care to improve well-being and quality of life. Given the growing number of long-term survivors, it is crucial to empower prostate cancer survivors to reduce psychological distress.

During the preparation of this work the authors used ChatGPT in order to check the English grammar and English words used. After using this tool/service, the authors reviewed and edited the content as needed and take full responsibility for the content of the publication.

## Data availability

The datasets analyzed during the current study are available from the corresponding author on reasonable request.

## CRediT authorship contribution statement

**Lilly J. Schmalbrock:** Conceptualization, Data curation, Writing – original draft, Visualization, Investigation, Writing – review & editing, Validation. **Nils Kager:** Data curation, Visualization, Investigation, Writing – review & editing. **Florian Kirchhoff:** Writing – review & editing, Data curation, Formal analysis. **Stefan Schiele:** Software, Validation, Writing – review & editing, Project administration. **Andreas Dinkel:** Conceptualization, Methodology, Writing – review & editing, Supervision. **Jürgen E. Gschwend:** Writing – review & editing, Project administration, Supervision. **Kathleen Herkommer:** Conceptualization, Methodology, Writing – original draft, Project administration, Supervision, Writing – review & editing.

## Declaration of competing interest

The authors declare no conflict of interest. No financial funding.
